# Supplementary material for: Common SNCA Genetic Variants and Parkinson’s Disease Risk: A Systematic Review and Meta-Analysis
Source: Int J Mol Sci. 2025 Jun 23;26(13):6001. doi: 10.3390/ijms26136001 (PMC12250285; doi:10.3390/ijms26136001)
Supplement: Supplementary file 1 [file ijms-26-06001-s001.zip › ijms-3634665-supplementary.pdf]

Table S1. Summary of Included Studies in the Meta-Analysis Investigating the Association Between SNCA Variants and Parkinson's Disease.

N	Study	Region	Sample Size	Age	Gender		PD type	SNP ID	Model type
			(Case/Control)	(Case/Control)	Case (M/F)	Control (M/F)			
1	Winkler, 2007	Germany and Serbia	Germany: 234/160<br>Serbia: 163/110	55.0±11.0/ 51.0±12.0	238/159	135/135	Idiopathic PD	rs356165	Allelic (G vs. A)
								rs2583988	Allelic (T vs. C)
								rs11931074	Allelic (T vs. G)
2	Myhre, 2008	Norway	236/236	58.2±11.4/ 69.7±10.9	140/96	140/96	PD	rs356165	Allelic (G vs. A)<br>Dominant (AG+GG vs. AA)<br>Recessive (GG vs. AG+AA)
								rs356219	Allelic (G vs. A)<br>Dominant (AG + GG vs. AA)<br>Recessive (GG vs. AA + AG)
								rs2583988	Allelic (T vs. C)<br>Dominant (TC+TT vs. CC)<br>Recessive (TT vs. TC+CC)
3	Guo, 2015	Han-Chinese	1019/1030	NA/52.16±16.4	423/596	490/540	Sporadic PD	rs356165	Allelic (G vs. A)<br>Dominant (AG+GG vs. AA)<br>Recessive (GG vs. AG+AA)
								rs11931074	Allelic (T vs. G)<br>Dominant (TG+TT vs. GG)<br>Recessive (TT vs. TG+GG)

4	Davis, 2016	PPMI (European) and Washington University in Saint Louis Movement Disorder Center (WU)	WU:418/306<br>PPMI:368/150<br><br>Total:456/786	WC:<br>NA/72.4± 15.1<br><br>PPMI:<br>NA/60.9±11.4	WU:258/160<br><br>PPMI:<br>240/128	WU: 105/201<br><br>PPMI:100/50	PD	rs356165	Allelic (G vs. A)
								rs356219	Allelic (G vs. A)
								rs11931074	Allelic (T vs. G)
5	Cardo, 2012	Spain	1169/772	59.0±12.0/<br>64.0±17.0	648/521	323/449	PD	rs356165	Allelic (G vs. A)<br>Dominant<br>(AG+GG vs. AA)<br>Recessive<br>(GG vs. AG+AA)
6	Ross, 2007	Ireland	186/186	61.0 ±12.0/<br>61.0 ±12.0	73/113	73/113	PD	rs356165	Allelic (G vs. A)<br>Dominant<br>(AG+GG vs. AA)<br>Recessive<br>(GG vs. AG+AA)
								rs11931074	Allelic (T vs. G)<br>Dominant<br>(TG+TT vs. GG)<br>Recessive<br>(TT vs. TG+GG)
7	Wu-Chou, 2013	Taiwan	626/473	NA/70.0± 6.8	NA	NA	Sporadic PD	rs356165	Allelic (G vs. A)<br>Dominant<br>(AG+GG vs. AA)<br>Recessive<br>(GG vs. AG+AA)
								rs356219	Allelic (G vs. A)<br>Dominant<br>(AG + GG vs. AA)<br>Recessive<br>(GG vs. AA + AG)

								rs11931074	Allelic (T vs. G)<br>Dominant<br>(TG+TT vs. GG)<br>Recessive<br>(TT vs. TG+GG)
8	Szwedo, 2021	Norway, Scotland, Swedish Cohort (2002-2009)	433/417	69.9±9.6/<br>69.6 ±10.2	263/170	242/175	Newly Diagnosed Patients	rs356219	Allelic (G vs. A)<br>Dominant<br>(AG + GG vs. AA)<br>Recessive<br>(GG vs. AA + AG)
9	Pan, 2012	Chinese Han	403/315	64.1 ± 9.0/<br>63.7±11.1	243/160	186/129	Sporadic PD	rs356219	Allelic (G vs. A)<br>Dominant<br>(AG + GG vs. AA)<br>Recessive<br>(GG vs. AA + AG)
10	Salas-Leal, 2021	Mexico	88/88	70.5 ± 9.4/<br>70.1 ± 9.3	46/42	46/42	PD	rs356219	Allelic (G vs. A)<br>Dominant<br>(AG + GG vs. AA)<br>Recessive<br>(GG vs. AA + AG)
11	Trotta, 2012	Italy	904/891	66.6±10.9/<br>62.4±14.6	543/361	308/583	PD	rs356219	Allelic (G vs. A)
								rs2583988	Allelic (T vs. C)
12	Wider, 2011	United States, Ireland, and Norway	1020/1095	71.0 ± 11.0/<br>71.0 ± 16.0	599/421	553/542	PD	rs356219	Allelic (G vs. A)<br>Dominant<br>(AG + GG vs. AA)<br>Recessive<br>(GG vs. AA + AG)
13	Goris, 2007	United Kingdom	649/2123	NA	NA	NA	PD	rs356219	Allelic (G vs. A)<br>Dominant<br>(AG + GG vs. AA)<br>Recessive<br>(GG vs. AA + AG)

14	Campelo, 2017	Brazil	105/101	64.4± 11.7/ 63.0 ± 10.0	73/32	68/33	PD	rs356219	Allelic (G vs. A) Dominant (AG + GG vs. AA) Recessive (GG vs. AA + AG)
								rs2583988	Allelic (T vs. C) Dominant (TC+TT cs. CC) Recessive (TT vs. TC+CC)
								rs11931074	Allelic (T vs. G) Dominant (TG+TT vs. GG) Recessive (TT vs. TG+GG)
15	Fernández-Santiago, 2019	Spain	898/921	Men: 64.4± 12.2, Women: 67.5± 11.3/Men: 53.8± 12.2, Women: 56.8± 14.3	511/387	539/382	PD	rs356219	Allelic (G vs. A) Dominant (AG + GG vs. AA) Recessive (GG vs. AA + AG)
16	Shahmohamm adibeni, 2015	Iran	489/489	59.31 ± 12.5/ 58.7 ± 12.2	260/229	258/231	PD	rS11931074	Allelic (T vs. G) Dominant (TG+TT vs. GG) Recessive (TT vs. TG+GG)
17	Emelyanov, 2018	Russia	458/353	64.4 ±10.3/ 61.7 ± 8.3	192/266	181/172	PD	rs2583988	Allelic (T vs. C) Dominant (TC+TT cs. CC) Recessive (TT vs. TC+CC)
								rs356219	Allelic (G vs. A) Dominant (AG + GG vs. AA) Recessive (GG vs. AA + AG)
								rs11931074	Allelic (T vs. G)

									Dominant (TG+TT vs. GG)
18	Heckman, 2012	United States	426/769	72.0± 11.0/ 79.0±11.0	233/193	385/384	PD	rs2583988	Allelic (T vs. C)
19	Li, 2013	China	685/569	NA/54.0± 13.0	395/290	306/263	Sporadic PD	rs356219	Allelic (G vs. A) Dominant (AG + GG vs. AA) Recessive (GG vs. AA + AG)
20	Miyake, 2013	Japan	229/357	68.4±8.7/ 66.6±8.5	88/141	138/219	Sporadic PD	rs356219	Allelic (G vs. A) Dominant (AG + GG vs. AA) Recessive (GG vs. AA + AG)
21	Yu, 2015	China	534/435	58.3 ± 11.0/ 52.3 ±14.1	315/219	244/ 191	Sporadic PD	rs356219	Allelic (G vs. A) Dominant (AG + GG vs. AA) Recessive (GG vs. AA + AG)
22	Liu, 2020	China	386/775	69.5 ± 14.6/ 68.3 ± 13.7	134/252	255/520	PD	rs356219	Allelic (G vs. A) Dominant (AG + GG vs. AA) Recessive (GG vs. AA + AG)
23	Chen, 2015	China	1276/846	60.4 ± 11.5/ 54.3± 12.8	709/567	476/370	PD	rs11931074	Allelic (T vs. G) Dominant (TG+TT vs. GG) Recessive (TT vs. TG+GG)
24	Satake, 2009	Japan	1078/2628	58.8 ±10.1/ 49.9 ±14.2	486/592	1439/1189	PD	rs11931074	Allelic (T vs. G)
25	Tan, 2010	China	433/916	Median: 64/56	242/ 191	550/ 366	PD	rs11931074	Allelic (T vs. G)
26	Hu, 2012	China	110/136	59.9± 10.5/ 59.9± 9.1	61/49	76/60	PD	rs11931074	Allelic (T vs. G) Dominant (TG+TT vs. GG)

									Recessive (TT vs. TG+GG)
27	Rahimi, 2017	Iran	500/500	60.8 ± 4.7/ 61.2 ± 5.3	268/232	273/277	Sporadic PD	rs11931074	Allelic (T vs. G) Dominant (TG+TT vs. GG) Recessive (TT vs. TG+GG)

Abbreviations: N: Number, PD: Parkinson's Disease; SNP: Single Nucleotide Polymorphism; M/F: Male/Female; NA: Not Available.
"Sporadic PD" refers to Parkinson's disease cases without a known family history, while "PD" includes both familial and sporadic cases or is not clearly specified in the original study.

Table S2. Quality assessment of included studies using the Newcastle-Ottawa Scale (NOS).

**Study**	**Selection**				**Comparability**		**Exposure**			**Total Score**
	**Case Definition Adequate**	**Representativeness of Cases**	**Selection of Controls**	**Definition of Controls**	**Control for Important Factor**	**Control for Additional Factor**	**Ascertainment of Exposure**	**Same Method of Ascertainment for Cases and Controls**	**Non-Response Rate**	
Winkler, 2007	✓	✓	✓		✓	✓	✓	✓		**7**
Myhre, 2008	✓	✓	✓				✓	✓		**5**
Guo, 2015	✓	✓	✓	✓	✓	✓	✓	✓		**8**
Davis, 2016	✓	✓	✓			✓	✓	✓		**6**
Cardo, 2012	✓	✓	✓	✓	✓	✓	✓	✓		**8**
Ross, 2007	✓		✓	✓	✓	✓	✓	✓		**7**
Wu-Chou, 2013	✓	✓	✓	✓			✓	✓		**6**
Szwedo, 2021	✓	✓	✓	✓	✓	✓	✓	✓	✓	**9**
Pan, 2012	✓	✓	✓	✓			✓	✓		**6**
Salas-Leal, 2021	✓	✓			✓	✓	✓	✓		**6**
Trotta, 2012	✓	✓	✓	✓	✓	✓	✓	✓		**8**
Wider, 2011	✓	✓	✓	✓	✓	✓	✓	✓		**8**
Goris, 2007	✓	✓	✓		✓	✓	✓	✓		**7**
Campelo, 2017	✓	✓	✓	✓	✓	✓	✓	✓	✓	**9**
Fernández-Santiago, 2019	✓	✓	✓	✓	✓	✓	✓	✓		**8**
Shahmohammadibeni, 2015	✓	✓		✓			✓	✓		**5**
Emelyanov, 2018	✓	✓	✓	✓	✓	✓	✓	✓		**8**

Heckman, 2012	✓	✓	✓	✓	✓	✓	✓	✓		**8**
Li, 2013	✓	✓	✓				✓	✓		**5**
Miyake, 2013	✓	✓	✓	✓	✓	✓	✓	✓	✓	**9**
Yu, 2015	✓	✓	✓	✓	✓	✓	✓	✓		**8**
Liu, 2020	✓	✓	✓	✓	✓	✓	✓	✓		**8**
Chen, 2015	✓	✓	✓				✓	✓		**5**
Satake, 2009	✓	✓	✓	✓			✓	✓		**6**
Tan, 2010	✓	✓	✓		✓	✓	✓	✓		**7**
Hu, 2012	✓	✓	✓	✓	✓	✓	✓	✓		**8**
Rahimi, 2017	✓	✓	✓	✓			✓	✓		6

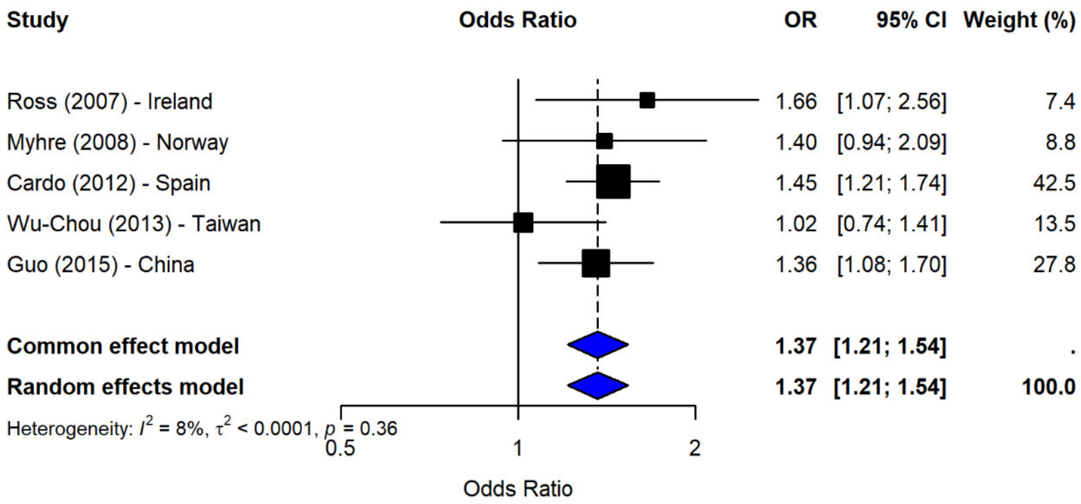

Figure S1. The association between SNP rs356165 and PD risk under the dominant model (GG+GA vs. AA).

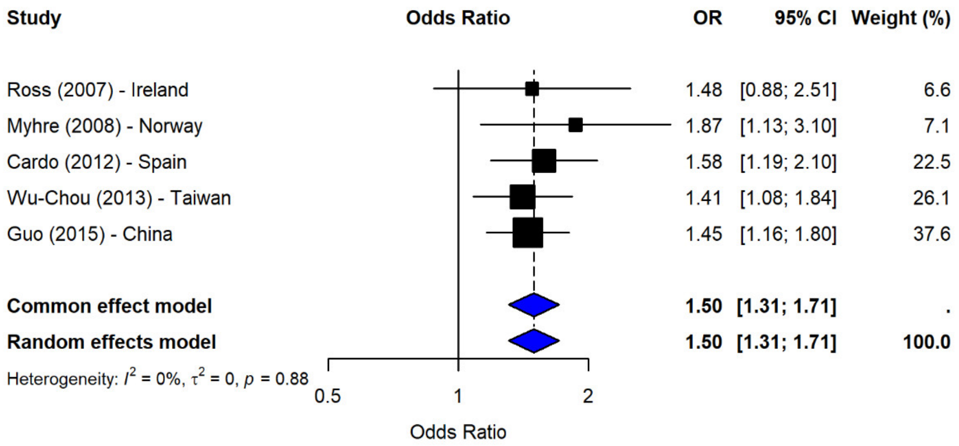

Figure S2. The association between SNP rs356165 and PD risk under the recessive model (GG vs. AG+AA).

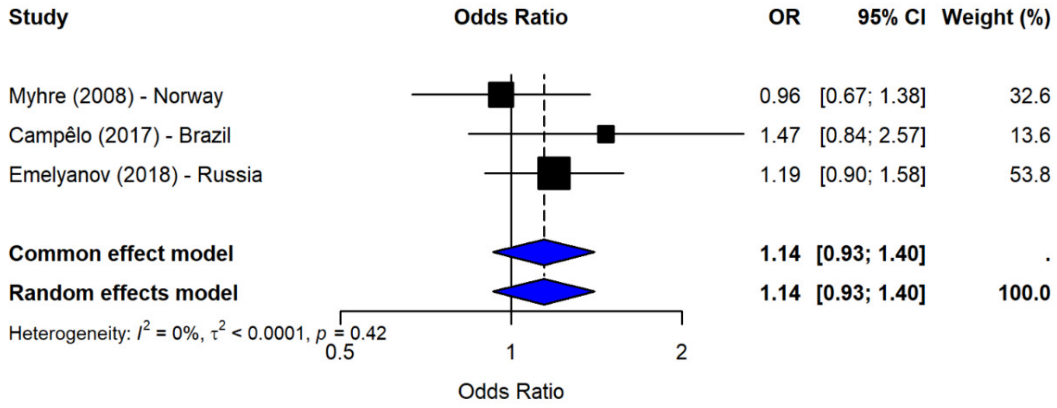

Figure S3. The association between SNP rs2583988 and PD risk under the dominant model (TT+TC vs. CC).

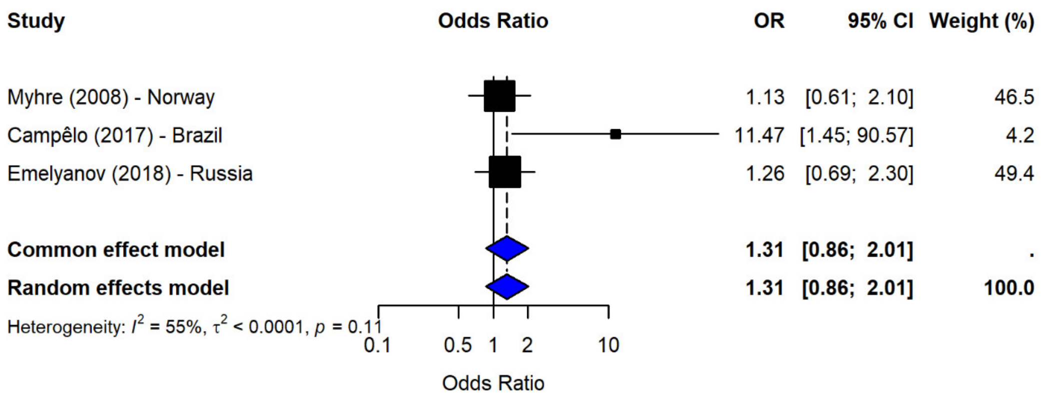

Figure S4 The association between SNP rs2583988 and PD risk under the recessive model (TT vs. TC+CC).

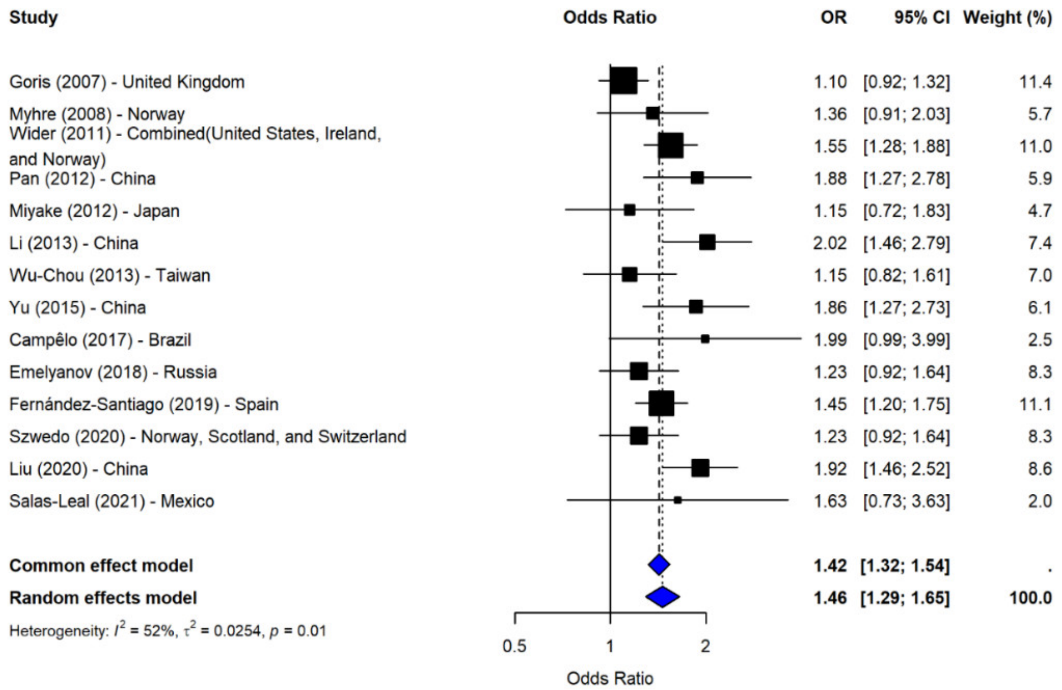

Figure S5. The association between SNP rs356219 and PD risk under the dominant model (AG+GG vs. AA).

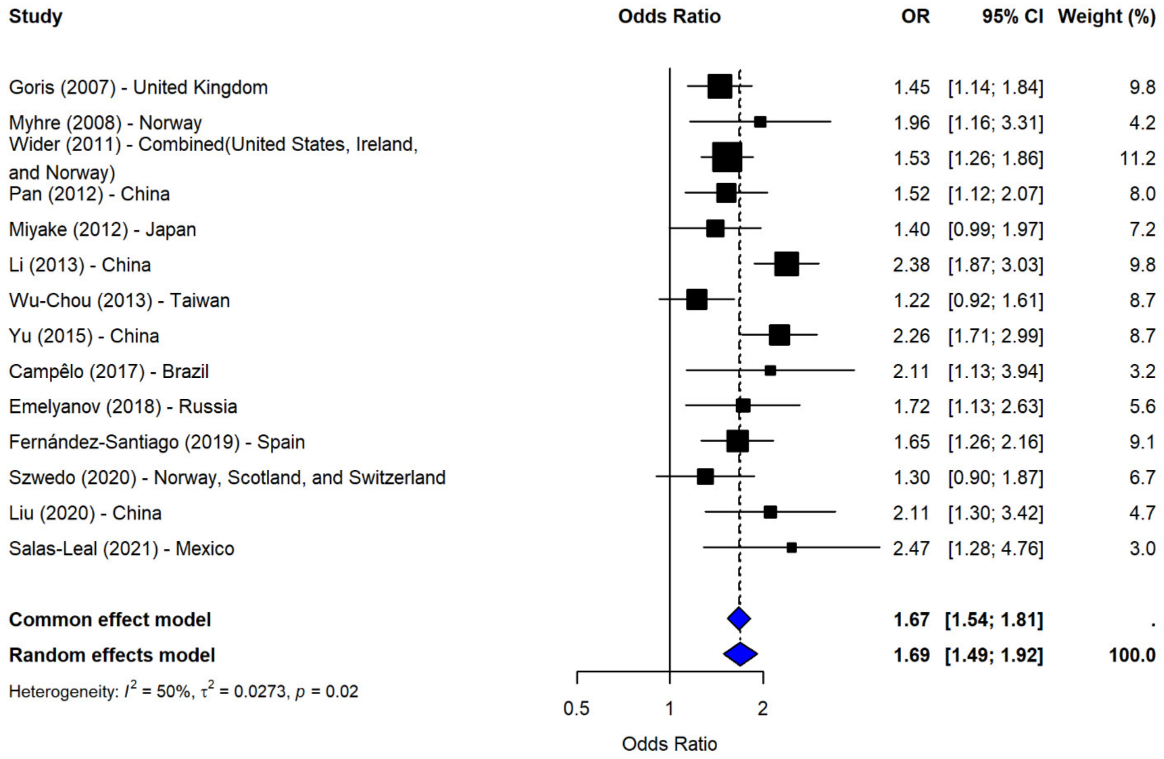

Figure S6. The association between SNP rs356219 and PD risk under the recessive model (GG vs. AG+AA).

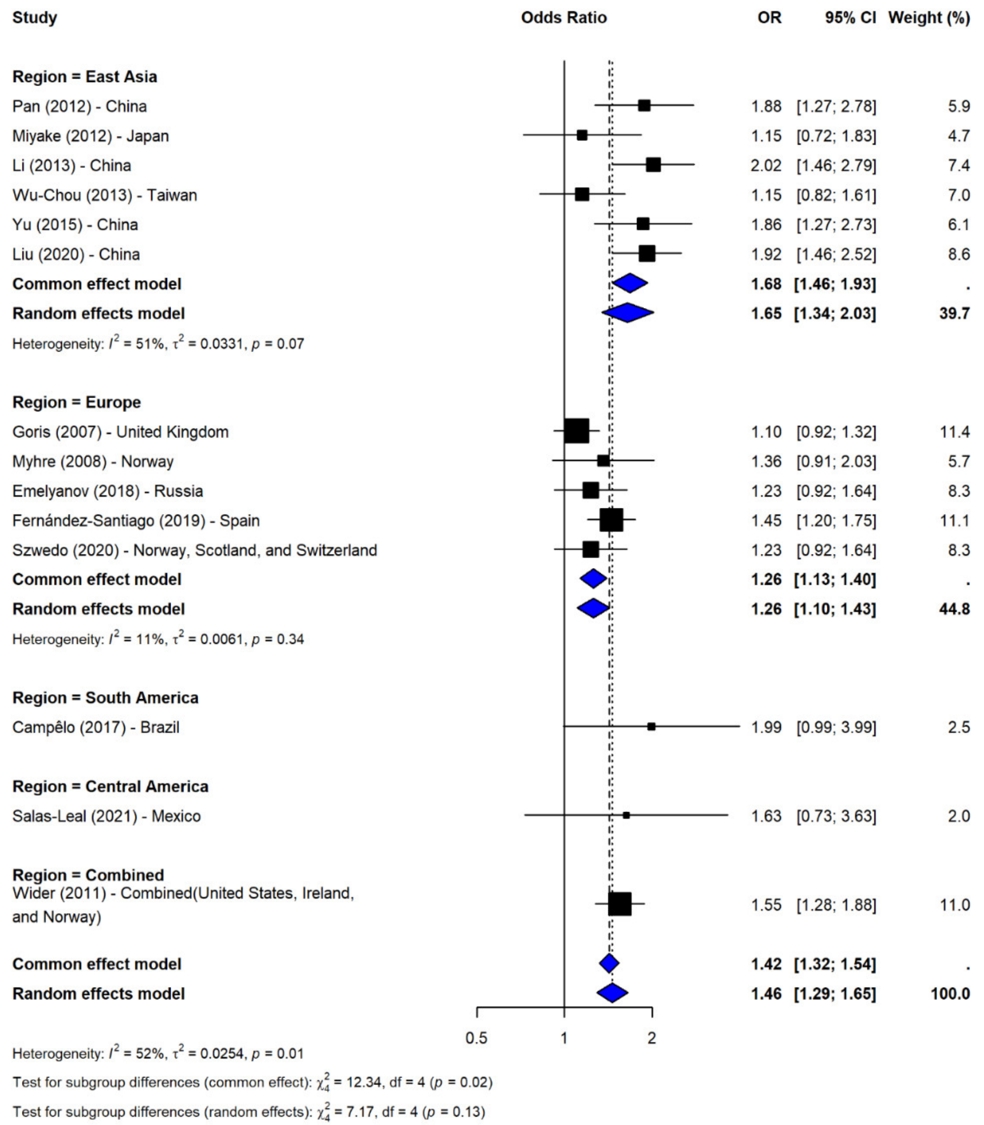

Figure S7. Subgroup meta-analysis of rs356219, under the dominant model (AG+GG vs. AA) by geographic region.

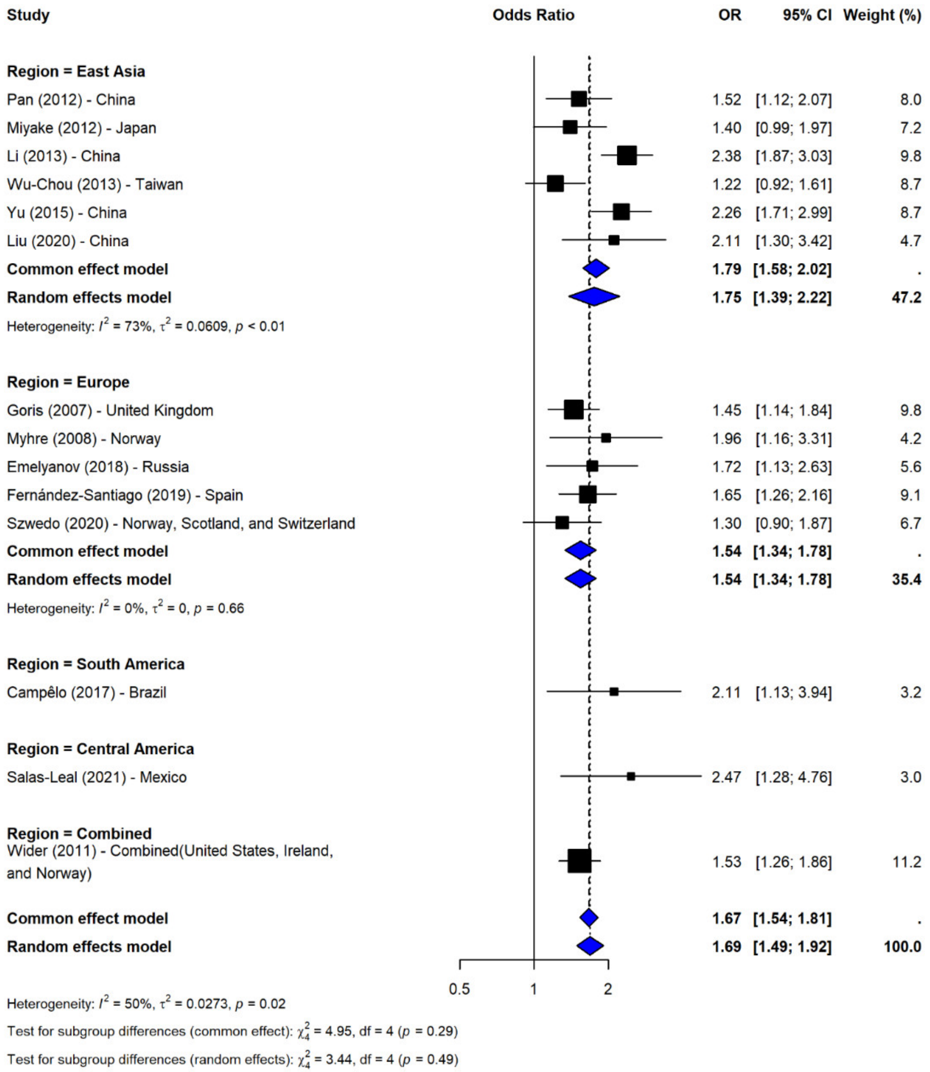

Figure S8. Subgroup meta-analysis of rs356219, under the recessive model (GG vs. AG+AA) by geographic region.
